# The effect of mortality salience and early-life maternal care on neuroendocrine, autonomic, and psychological stress responses

**DOI:** 10.1038/s41598-025-85380-w

**Published:** 2025-01-08

**Authors:** Ulrike U. Bentele, Paula Strobel, Maria Meier, Annika B. E. Benz, Raphaela J. Gaertner, Elea S. C. Klink, Bernadette F. Denk, Stephanie J. Dimitroff, Eva Unternaehrer, Jens C. Pruessner

**Affiliations:** 1 Department of Psychology, Division of Neuropsychology, University of Constance, Fach 905, Universitaetsstrasse 10, 78464 Constance, Germany; 2 Centre for the Advanced Study of Collective Behaviour, University of Constance, Constance, Germany; 3 Child- and Adolescent Research Department, University Psychiatric Clinics Basel (UPK), University of Basel, Basel, Switzerland; 4 Department of Psychology, University of Montana, Montana, 59812 USA

**Keywords:** Early-life adversity, Maternal care, Mortality salience, HPA axis, Stress, TSST, Stress and resilience, Biomarkers

## Abstract

Adverse early-life experiences alter the regulation of major stress systems such as the hypothalamic-pituitary-adrenal (HPA) axis. Low early-life maternal care (MC) has repeatedly been related to blunted cortisol stress responses. Likewise, an acutely increased awareness of mortality (mortality salience [MS]) also has been shown to blunt cortisol responses. In this study we investigated the effects of early-life MC and a potential interaction with MS on HPA axis responsivity, as well as autonomic and subjective stress responses. Seventy-three women (*M*_*age*_=21.56, *SD*_*age*_=2.85) with self-reported low (*n* = 30) or high (*n* = 43) early-life MC, underwent the Trier Social Stress Test for groups. Before, they were asked to briefly contemplate either death (mortality condition, *n* = 38) or sleep (control condition, *n* = 35). Salivary cortisol and alpha amylase, heart rate variability and subjective stress levels were assessed repeatedly. Multilevel mixed models confirmed an effect of MC on stress system regulation, indicated by blunted cortisol responses and overall reduced heart rate variability in low versus high MC individuals. Moreover, we found an interaction between MS and MC concerning subjective stress and autonomic measures. Specifically, low MC individuals in the control compared to the mortality condition showed both overall higher subjective stress levels, and less increase in heart rate variability following stress. These findings demonstrate the enduring impact of low early-life MC and the potential role of acute mortality primes on the regulation of stress systems in healthy women.

**Supplementary Information:**

The online version contains supplementary material available at 10.1038/s41598-025-85380-w.

## Introduction

Acute psychosocial threat triggers an integrated response of multiple biological systems^1,2^. Activation of the autonomic nervous system (ANS), reflected in fast parasympathetic (PNS) withdrawal and sympathetic (SNS) dominance, is thereby followed by an activation of the hypothalamic–pituitary–adrenal (HPA) axis. Cortisol as the primary end product of the HPA axis mediates adaptive metabolic, cardiovascular and reproduction processes^3^. While adequate stress responses promote survival and health^4^, dysregulated exaggerated (hyper-) or blunted (hypo-) responses are related to an increased risk for diseases^5,6^. Chronic exposure to early-life adversity (ELA) has been identified as a major factor that impacts stress system regulation.

## Early-life adversity and HPA axis reactivity

ELA comprises a wide range of adverse or stressful experiences during childhood and adolescence^7,8^, that are characterized by the perception of threat or unpredictability^9^. In the past, ELA has thus been studied and operationalized in manifold ways. Frequently considered forms of ELA include traumatic events (e.g., exposure to death or abuse)^10^. Increasingly, measures of dysfunctional child-caregiver relationships are studied (e.g., parental mental illness/separation, a low extent of maternal caring)^11,12^. Specifically, the variable employed in the current study, low early-life maternal care (MC), is considered one form of ELA, with lower MC indicating higher ELA.

Extensive empirical work has linked ELA to persistent alterations in HPA axis reactivity^13^, mainly evident in blunted cortisol stress responses in adults exposed to ELA^14,15^. Findings on maternal caregiving confirmed more reduced cortisol responses to the Tier Social Stress Test (TSST^16^), in individuals with low compared to medium early-life MC^17^; however, findings remain inconsistent (e.g., ^18^). Following evolutionary-developmental theories^19,20^, reactivity of the stress response systems fulfills an evolutionary adaptive function. It is involved in the development of traits and behaviors that allow to achieve major life functions, such as reproduction (e.g., the Adaptive Calibration Model [ACM])^19,21^. In adverse environments, which signal potentially shortened lifetime, blunted HPA axis reactivity is thereby considered to promote earlier maturation or reproduction, so-called fast life history traits^21^. Taken together, exposure to low early-life MC may be linked to blunted HPA axis stress responses in adulthood. Such dysregulated stress responses are considered to contribute to an increased risk for various physical and mental disorders (e.g.,^22,23^). Specifically, chronic activation of the stress system and release of cortisol unfolds various cellular effects, and thus leads in the long run to a wear out of the stress system^24^. Failure to show a response when needed may result in an over-reaction of other systems (e.g., inflammation) that play a central role in overall health^25^.

### Mortality salience and HPA axis reactivity

The instinctive human drive for survival contrasts with the natural end of life^26,27^. Awareness of the inevitability of death (mortality salience [MS]) thus constitutes a state of existential threat^28,29^, that elicits uncertainty or uncontrollability^30^. A common method to experimentally manipulate MS is to increase conscious death thoughts by contemplating death (MS stimulus), compared to contemplating a control topic (control stimulus [CS])^26^.

Previous work has extensively demonstrated the effects of MS on behavioral and cognitive outcomes^26^, e.g., an increased desire for offspring^31^. Though MS is theorized to also affect physiological systems^30,32^, empirical work regarding the effect of MS on ANS and particularly HPA axis activation is scarce (e.g.,^33^). First studies have shown that MS might affect HPA axis responses to subsequent stress^34–36^. Specifically, young males (aged 18 to 27 years) who had contemplated death (MS) compared to dental pain (CS) showed reduced cortisol responses to a subsequent social stress task (i.e., watching an out-group stress video)^35^. This finding was surprising as considering one’s own death was hypothesized to intensify physiological responses to a stressor. One explanation could be the evolutionary function of cortisol (e.g., for reproduction) under conditions of existential threat, as outlined in the ACM^19^. Under conditions that signal premature mortality and unpredictability, the time to grow and reproduce before dying is short. Organisms therefore develop strategies that allow them to live faster, e.g., show earlier age of maturation or first reproduction. The HPA axis and cortisol are part of this fast life-history strategy. As cortisol contributes to the inhibition of reproductive functions (e.g.,^37^). low cortisol levels support reproductive functions. Thus, existential threats, such as an increased awareness of one’s own mortality (e.g., MS), might lead to reduced cortisol responses to stress in the short term. However, it remains open whether the effect of MS manipulations might vary with past experiences of threat early in life, such as ELA.

## ELA, mortality salience and HPA axis reactivity

From these separate lines of research, the question arises whether acute MS and chronic exposure to ELA might converge in their neuroendocrine effects. Specifically, we were interested in whether ELA and MS might trigger similar processes and thus subsequent biobehavioral responses, such as HPA axis hyporesponsivity: In this sense, chronic exposure to ELA has mainly been linked to blunted cortisol stress responses in healthy adults (e.g.,^14^). while there is some evidence that exposure to mortality primes could lead to reduced cortisol stress responses, as well (e.g.,^35^). The processes leading to such hyporesponsive cortisol patterns are not fully understood. Against this background, we wondered whether MS might for a limited period of time trigger a similar process or state also in healthy individuals without ELA, that emerges from chronic exposure to ELA. Empirically, an acute MS manipulation might then temporarily mimic the enduring effects of chronic early-life stress (i.e., cortisol hyporesponsivity to a stressor) in individuals without ELA; MS would not affect cortisol stress responses in individuals with ELA. In line with this conceptual idea, previous work has pointed to high conceptual similarities between the constructs of MS and ELA. Both present conditions of existential threat^19,27^, that imply high unpredictability or uncertainty. Both signal premature mortality or death, and are thus operationalized by indicators of mortality (e.g., parental death, death thoughts). Moreover, both exposure to ELA and MS have been linked to alterations in brain regions^30,38^, that are specifically involved in regulation of the stress system, including activation of the HPA axis^39^. Finally, there is evidence for the proposed interaction between MS and ELA on HPA axis responses^36^. Healthy, young males (aged 18–35 years; no mental illness; body-mass index [BMI] 18 to 27 kg/m^2^) with a low or high history of ELA first contemplated death (MS) or sleep (CS) before they underwent a psychosocial stress task. MS compared to CS resulted in blunted cortisol responses to the stressor in low ELA males; no effect of MS occurred in high ELA males. However, the results are limited to male sex and HPA axis reactivity, and thus warrant further clarification. Sex might moderate the effects of ELA^40^ and MS^35^. For ELA, females rather than males may exhibit more blunted HPA axis responses following ELA^14^. A possible explanation is that women differ from men in the type of ELA they experience (e.g., more sexual abuse), as well as the frequency of exposure (e.g., longer duration), which would affect the development of HPA axis dysregulation. For MS, women compared to men might use protective strategies to defend themselves when exposed to death (e.g., habitual mindfulness, religion). Since we did not have sufficient resources to recruit and test a mixed-sex sample, we focused on a comparable (e.g., with respect to age, health status) but female sample only. Moreover, assessment of multiple stress systems is recommended for an adequate investigation of stress reactivity (e.g.,^41^). , which is why we also assessed ANS and subjective measures.

Thus, the aim of the current study was twofold. First, we sought to investigate the effect of early-life *MC* and *MS* on HPA axis responses to psychosocial stress in young females, aged 18 to 30 years. We chose a sample of a young, narrow age range to eliminate potential age effects on cortisol stress responses as recommended (see methods); moreover, we decided for this specific age range to keep our sample comparable to prior studies (18 to maximum 35 years, e.g.,^17,36^). This also has drawbacks. Participants’ age could also change the impact of early-life MC experiences on adult stress responses (e.g., the older, the less impact). Similarly, age, and therefore proximity to one’s own death, could also alter the impact of MS interventions on the stress response (e.g., the older, the stronger or the less impact). Therefore, the results cannot be generalized to other age groups. For this, healthy women were screened for self-reported MC during early life (high versus low), using the corresponding subscale and cut-off value from the Parental Bonding Instrument (PBI)^42^. Next, they underwent a MS manipulation paradigm, that included to contemplate their own death (MS condition) or sleep (control condition) for ten minutes. Finally, participants were exposed to the TSST for groups (TSST-G)^43^. We assumed (1) that MC was related to cortisol stress responses, indicated by more blunted responses in low versus high MC individuals (H1), and (2) an interaction between MC and MS on cortisol stress responses, indicated by blunted responses in high MC individuals following death (MS) versus sleep contemplation (CS) (H2). Second, we extended prior work by exploring the effect of MC and MS on subjective and autonomic stress responses. Changes in salivary alpha-amylase (sAA) were thereby considered to primarily reflect responses of the SNS, changes in heart rate variability (HRV), specifically respiratory sinus arrythmia (RSA), to reflect changes of PNS activity.

## Results

### Sample characteristics

The final sample included *n* = 22 (high MC & MS), *n* = 21 (high MC & CS), *n* = 16 (low MC & MS) and *n* = 14 (low MC & CS) women (*M*_*age*_=21.56, *SD*_*age*_=2.85, range: 18–30 years). Groups differed regarding (1) age (*F*(3,69) = 5.72, *p* = .001, η^2^ = 0.199), indicating younger age in the high MC & MS group compared to both low MC groups (*p*s < = 0.018), (2) depressiveness (*F*(3,69) = 3.25, *p* = .027, η^2^ = 0.124), indicated by higher Beck’s Depression Inventory (BDI) sum scores in the low compared to the high MC groups (*p*s < = 0.048), (3) duration of daily physical exercise (*F*(3,69) = 3.96, *p* = .012, η^2^ = 0.147), indicating longer exercising in the high MC & CS compared to the low MC groups (*p*s < = 0.032), and (4) cohort (*p* < .001) with low MC groups being tested exclusively in the second post-pandemic wave. For group comparisons see Table 1.

**Table 1:** Participant characteristics and comparisons of the four experimental groups (*N* = 73).

Mortality condition	Maternal care				*p* ^a^
	High		Low		
	MS	CS	MS	CS	
*n*	22	21	16	14	
PBI maternal care	33.32(2.03)	33.52(2.27)	18.06(7.25)	20.86(6.49)	**< 0.001**
Age [yr]	19.91(1.72) range: 18–25	21.38(2.36) range: 19–29	23.13(3.12) range: 20–30	22.64(3.39) range: 18–30	**0.001**
BMI [kg/m^2^]	22.16(2.57)	21.66(2.24)	22.30(1.61)	21.06(3.01)	0.459
Hormonal status (OC/FP/LP/UP)	10/6/2/4	10/3/4/4	4/7/3/2	3/5/2/4	0.568
Cohort (prior/post pandemic)	22/0	21/0	8/8	9/5	**< 0.001**
Depressiveness	5.18(4.11)	5.43(3.57)	8.50(6.06)	8.93(4.78)	**0.027**
Self esteem	23.27(4.34)	23.14(4.34)	20.25(4.61)	20.14(4.54)	0.052
Physical exercise [min/day]	50.45(33.56)	79.43(63.69)	39.26(27.38)	37.50(22.60)	**0.012**
Sleep duration [min]	500.77(67.55)	474.76(91.03)	502.88(63.66)	480.36(64.40)	0.558
Religious identification	0.86(0.94)	0.95(1.12)	0.81(1.22)	1.14(1.29)	0.859

Mean values (± standard deviations) or absolute frequencies of participant characteristics. Religious identification was assessed using one item asking for subjective religiosity to be answered from 0 (not at all) to 4 (completely). Self-esteem was assessed using the Rosenberg Self-Esteem Scale. Depressiveness was measured using the Beck Depression Inventory II. Significant differences are displayed in bold. MS = mortality salience, CS = control stimulus, BMI = body mass index, FP = follicular phase, LP = luteal phase, UP = unclear phase, OC = use of oral contraceptives, PBI = Parental Bonding Instrument.

^a^ p-values result from one-way ANOVAs (age, BMI, PBI, depressiveness, self-esteem, physical exercise, sleep duration, religiosity), or Fisher’s exact test (hormonal status, cohort; > 20% cells with expected frequencies smaller than 5).

### MS manipulation check

Following the MS manipulation, participants answered a Word fragment Completion Taks (WCT; for details see methods section on manipulation check). The task included word fragments that could be completed to either neutral or death-related words. A higher number of words completed with respect to death (WCT score), indicated a more successful MS induction. A Kruskal-Wallis test comparing the WCT scores confirmed the successful induction of death thoughts by MS, *H*(1) = 6.24, *p* = .012. In the MS group, individuals completed more word fragments in relation to death compared with the CS group (MS: *M* = 1.02, *SD* = 0.82; CS: *M* = 0.57, *SD* = 0.70).

### Effect of MC and mortality salience on cortisol stress responses

The effects of MC and MS on cortisol responses were assessed using hierarchical growth curve models (GCM), comprising the step-wise inclusion of random and fixed effects, e.g., of time, MC and MS (for details, see section on statistical analyses). Cortisol trajectories were best explained by a GCM with random intercepts, random slopes across participants, fixed linear, quadratic, and cubic *time* trends and a covariance structure (unconditional model). Inclusion of *MC* by *time* interactions (H1 model) significantly increased model fit; inclusion of *MC* by *mortality* by *time* interactions (H2 model) did not lead to model improvement (see supplemental material, S1 and S2). The interpretation of the results did not change when including MC as continuous predictor (see supplemental material, S3).

**Fig. 1:**
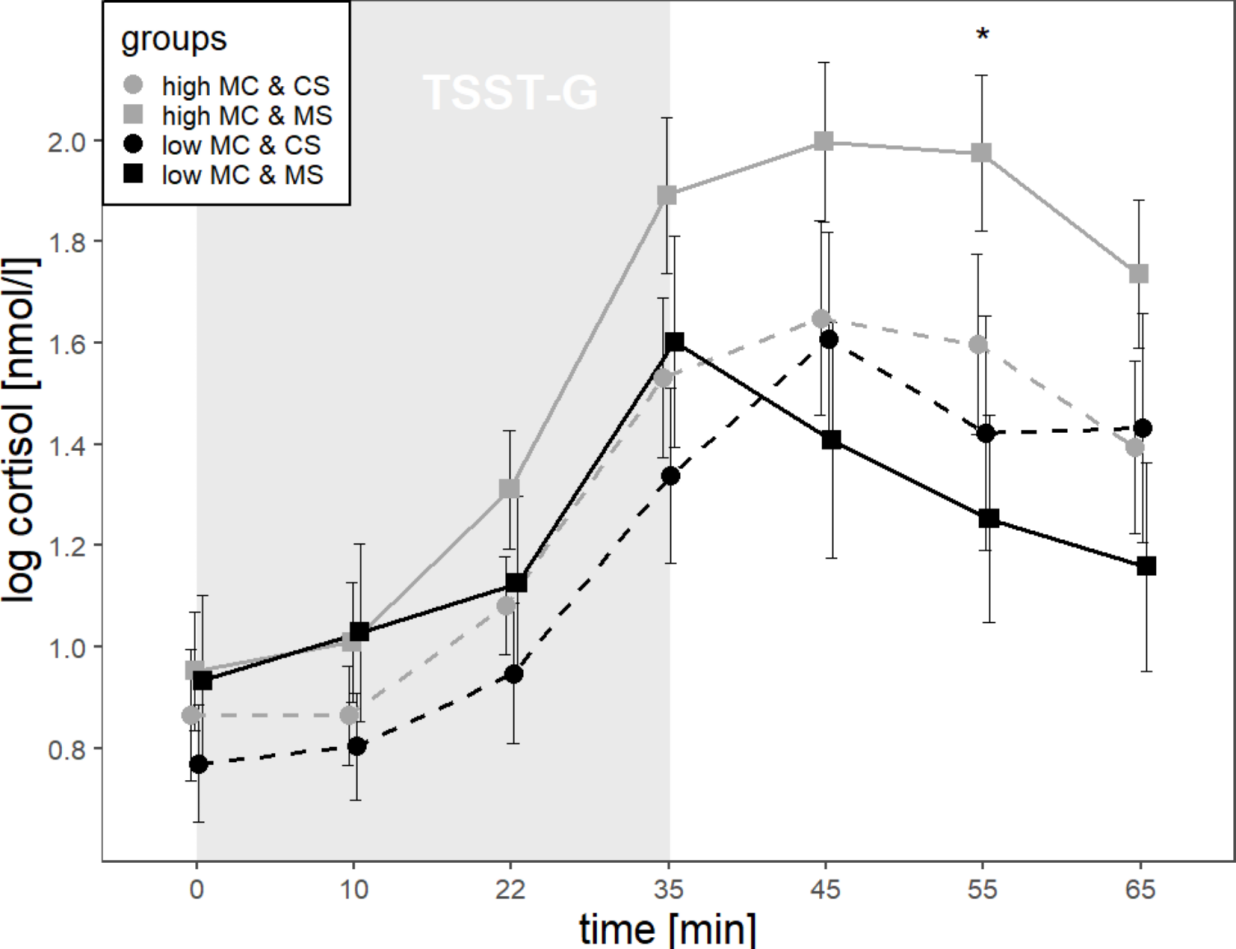
Changes in cortisol levels (logarithmised) over time in the four groups that vary with regard to MC (low, high) and MS (MS, CS). Values represent means *± SE*. There was a significant time by MC interaction: Individuals with low compared with high MC showed reduced cortisol stress responses, indicated by lower cortisol levels during parts of the recovery phase (*p* = .019). TSST-G = Trier Social Stress Test for groups, MC = maternal care, MS = mortality salience, CS = control stimulus.* *p* < = 0.05.

The final model revealed a significant main effect of *time* (*F*(3,432) = 52.63, *p* < .001, f = 0.60), and a *time* by *MC* interaction (*F*(3,432) = 2.65, *p* = .048, f = 0.14); the main effect of *MC* was not significant (*F*(1,71) = 0.87, *p* = .353, f = 0.11). Post-hoc tests indicated that cortisol levels increased during the TSST-G (between t = + 10 and t = + 35; *p*s < = 0.001) with peak levels ten minutes after stressor ending (t = + 45: *M* = 1.69, *SD* = 0.84), and a decline during recovery (t = + 55 to t = + 65; *p* = .001). However, within the low MC group individuals showed flattened trajectories, indicated by delayed and reduced cortisol increases (t = + 22 to t = + 35; *p* < .001) and no decreases during recovery (between t = + 35 and t = + 65; *p*s > = 0.053), as illustrated in Fig. 1. We also examined the potential effect of covariates (listed in the section on psychological measures). For this, we tested whether adding a singular covariate and the covariate by time interaction to the unconditional model significantly increased model fit. Inclusion of age, religious identification and self-esteem led to an improvement in model fit. In the best model that included *MC* and *MC* by *time* interactions as predictors, but controlled for *age* or *religious identification*, interpretation of the results with a significant effect of *time* and *time* by *MC* interaction did not change. In the model that controlled for *self-esteem*, none of the previous main or interaction effect of interest remained significant.

### Effect of MC and mortality salience on subjective and autonomic responses

#### Subjective-emotional stress response

Subjective stress trajectories were best described by a model with random intercepts, random slopes, fixed linear, quadratic, and cubic *time* trends and a covariance structure (unconditional model). The inclusion of a *MC* by *mortality* interaction further increased model fit significantly (see supplemental material, S4).

**Fig. 2:**
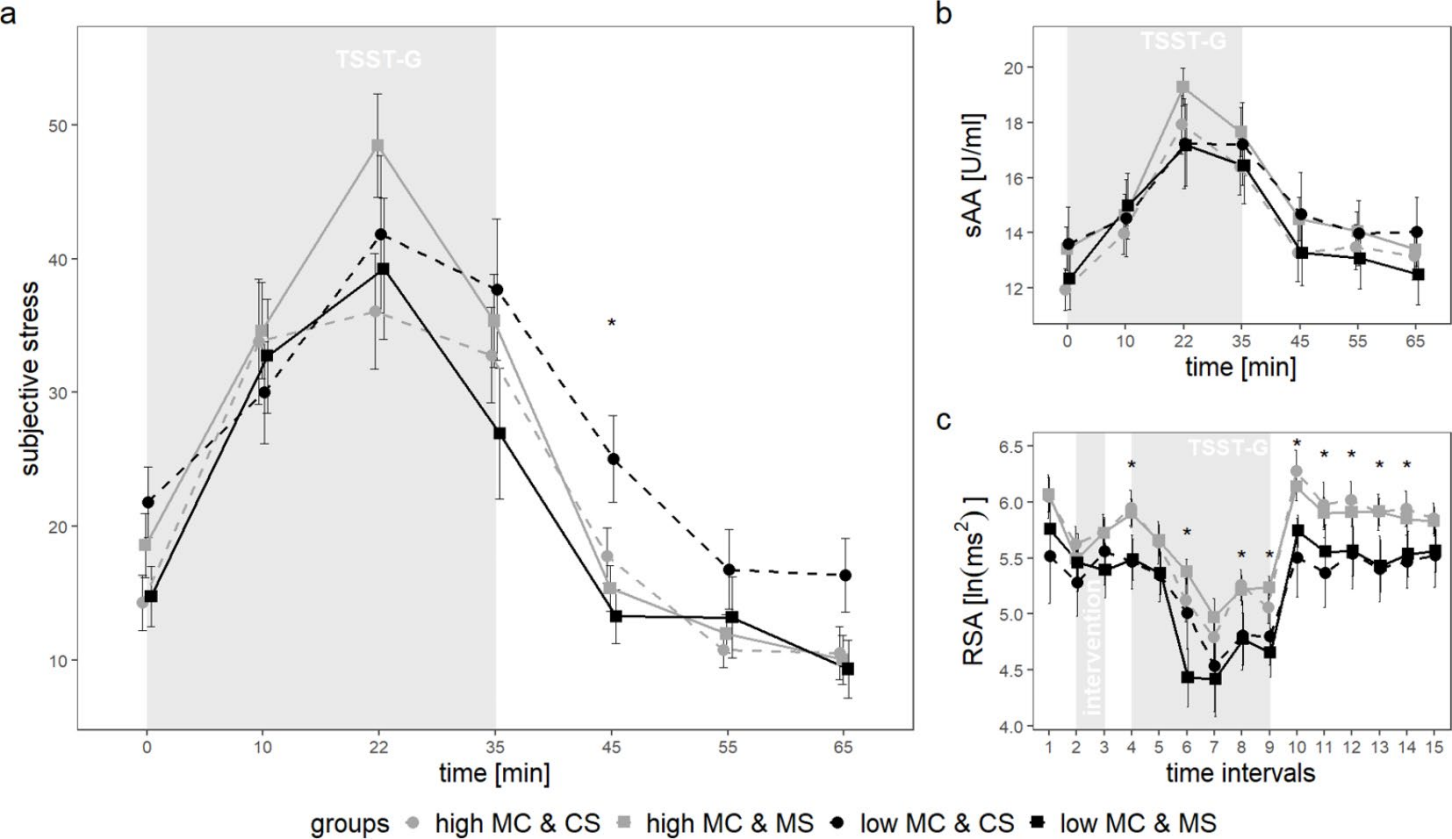
Changes in (**a**) subjective stress, (**b**) sAA (root transformed) and (**c**) RSA levels over time in the four groups that vary with regard to MC (low, high) and MS (MS, CS). Intervention refers to the experimental MS manipulation. Values represent means ± *SEs*. For subjective stress (**a**) we found a significant MC by MS interaction: Low MC individuals in the CS compared with the MS condition showed higher subjective stress levels, particularly during the recovery phase (*p* = .004). For RSA (**c**) there was a significant main effect of MC: Individuals with low compared with high MC showed an overall lower RSA, particularly during the stress and recovery phase (*p*s < = 0.039). Significant group differences (in a, c) are marked with an asterisk. MC = maternal care, MS = mortality salience, TSST-G = Trier Social Stress Test for groups, sAA = salivary alpha amylase, RSA = respiratory sinus arrhythmia. * *p* < = 0.05.

Evaluation of this final model showed a significant effect of *time* (*F*(3,429) = 98.39, *p* < .001, f = 0.83) and a *mortality* by *MC* interaction (*F*(1,69) = 4.96, *p* = .029, f = 0.27). No other main or interaction effects were significant (*F*s < = 1.44, *p*s > = 0.231). Post-hoc tests indicated that across groups subjective stress levels increased during the anticipation phase until the middle of the TSST-G (between t = 0 to t = + 22; *p*s < = 0.003; peak levels at t = + 22: *M* = 41.63, *SD* = 20.23) and decreased during recovery (between t = + 35 and t = + 55; *p*s = < 0.001). To follow up on the *mortality* by *MC* interaction, the effect of *mortality* was tested within each *MC* group separately using two analogue growth curve models. Results revealed a significant main effect of *time* (*F*(3,174) = 24.95, *p* < .001, f = 0.66) and *mortality* (*F*(1,28) = 4.86, *p* = .036, f = 0.42) in the low MC group only. Low MC individuals in the control compared to the mortality group perceived higher subjective stress, depicted by the black lines in Fig. 2a. Those low MC individuals in the control versus the mortality group particularly differed in subjective stress during the recovery phase (t = + 45; *t*(28) = 3.17, *p* = .004). They did not differ in any other timepoint (*p*s > 0.052).

#### sAA stress response

Trajectories of sAA levels were best explained by a model with random intercepts, fixed linear, quadratic, and cubic *time* trends and a covariance structure (unconditional model). Hierarchically adding *MC* and *mortality* and interactions, as well as possible covariates did not increase model fit (see supplemental material, S5).

The final model showed a main effect of *time* (*F*(3,405) = 67.42, *p* < .001, f = 0.71), displayed in Fig. 2b. Overall, sAA levels increased until the middle of the TSST-G (between t = 0 and t = + 22; *p*s < 0.001; peak levels at t = + 22: *M* = 18.03, *SD* = 4.79), before sAA levels immediately decreased following TSST-G cessation (t = + 35 to t = + 45; *p* = < 0.001).

#### RSA response

RSA trajectories were best described by a model with random intercepts, fixed linear, quadratic, and cubic *time* trends and a covariance structure (unconditional model). Adding *MC* further increased model fit (see supplemental material, S6).

Model evaluation revealed a main effect of *time* (*F*(3,977) = 30.20, *p* < .001, f = 0.30) and *MC* (*F*(1, 68) = 6.22, *p* = .015, f = 0.30). Regarding the *time* effect, post-hoc tests showed a significant RSA decrease from resting to MS intervention (I_1_ to I_2_; *p* = .008), and during TSST-G anticipation and speech task (between I_4_ and I_6_; *p*s < = 0.026); RSA increased again from the stress to the recovery phase (I_9_ to I_10_; *p* < .001). The main effect of *MC* indicated that high compared with low MC individuals exhibited overall higher RSA levels (high MC: *M* = 5.68, *SD* = 0.78, low MC: *M* = 5.28, *SD* = 1.05). Specifically, individuals with low versus high MC differed in RSA levels during the initial phases of TSST-G anticipation (I_4_; *t*(68)=-2.64, *p* = .010) and the speech task (I_6_; *t*(68)=-2.60; *p* = .011), as well as the recovery phase (I_8_-I_14_; all *p*s = < 0.039). RSA trajectories as displayed in Fig. 2c. Subsequent models that controlled for significant effects of *sleep duration* or *physical exercise* did not show changes of significant results. When *age* was controlled, the effect of MC was no longer significant.

Regarding RSA recovery, a two-way ANOVA revealed a significant *MC* by *mortality* interaction (*F*(1,66) = 4.43, *p* = .039, η^2^ = 0.062); neither the main effect of *mortality* nor *MC* was significant (*F*s < = 0.56, *p*s > = 0.457). Low MC individuals in the control compared to the mortality group exhibited less RSA increase following stress (CS: *M* = 0.72, *SD* = 0.91, MS: *M* = 1.17, *SD* = 0.74), though post-hoc t-tests did not indicate significant group differences. Concerning RSA reactivity, there were no significant main nor interaction effects (*F*s < = 1.62, *p*s > = 0.207).

## Discussion

The aim of the current study was to test the effect of ELA, operationalized as low early-life MC, and the interaction between ELA and MS on cortisol and psychophysiological stress responses in healthy, young females. For this purpose, women with high or low MC were exposed to the TSST-G, after they had either contemplated death (MS) or sleep (CS). The principal findings showed, firstly, that MC affected cortisol stress responses, indicated by blunted responsivity in low compared to high MC individuals. Secondly, MC and MS did not interact in predicting cortisol stress responses, while interactions between MC and MS were found with regard to autonomic (RSA) and subjective stress measures.

### Link between early-life adversity, operationalized by low early-life MC, and stress reactivity

The findings are in line with and expand on previous research, that has linked exposure to ELA with blunted HPA axis reactivity in adulthood^14,15^. Yet, some studies also found HPA axis hyperreactivity following ELA^13^. Two explanations might account for this inconsistency. Firstly, operationalizations of ELA broadly differ between studies^15,44^. Recent meta-analyses revealed that HPA axis reactivity was not related to ELA, when operationalized as abuse and neglect^45^; however, HPA axis hyporesponsivity was found, when ELA was operationalized more broadly including maternal caregiving behavior^14,15^. Thus, those different types of ELA might be linked to different alterations in HPA axis regulation in adulthood. Recent conceptual models suggest, that all types of ELA share common characteristics, such as the perception of threat, unpredictability or deprivation (e.g.,^46^). Different ELA types might, however, differ in which characteristics dominate. Low MC might be characterized by high deprivation (e.g., lack of stimulation) and unpredictability (e.g., lack of consistent supervision), whether abuse might be characterized by a higher degree of threat (e.g., to physical integrity). Those differences might then determine variations in the outcome. Here, we found that low MC was linked to HPA axis dysregulation in a similar way to other ELA types such as abuse. Operationalizations of ELA not only differ with respect to the form of ELA, but also differ whether they are based on objective or subjective measures. Recent work pointed to the significance of the subjective experience of ELA^11,47^. In line, it was found that the subjective experience of adversity (e.g., as measured by self-report) rather than the pure objective exposure to it (e.g., official records) confers an increased health risk^48^. Here, we correspondingly found HPA dysregulation to be linked to a subjective measure of ELA, i.e., self-reported perception of MC. Second, variations in participants’ sex might contribute to the heterogenous findings. Sex has been found to moderate the effect of ELA on HPA axis regulation^14,45^. As effects of ELA have mostly been examined in males, the effects in females remain more unclear^40^. Thus, the current study complements prior work in these aspects, showing that the subjective perception of low early-life MC might also link to HPA axis hyporeactivity in young adult females.

Our results further revealed an effect of ELA, operationalized by low MC, on PNS activation, indicated by overall reduced HRV in low compared to high MC individuals. This is consistent with the majority of previous studies that have linked ELA with reduced tonic HRV in older adults^44^. While high HRV is considered to index self-regulation and adaptability to environmental changes^49^, low HRV has been related to psychopathology (e.g.,^50^).

Together, these findings highlight the impact of ELA on *multi*systemic stress responsivity. This can both be understood from a theoretical and mechanistic perspective. According to developmental theories, multiple stress systems and their responses serve the joint function to enable adaptation and survival in changing environments^19,23^. The adaptive calibration model (ACM)^19^ proclaims distinct profiles of stress reactivity, that develop depending on environmental conditions. Severe ELA is related to a hyporesponsive profile, indicated by blunted SNS, PNS and HPA (re)activity. Blunted HPA axis responsivity and cortisol release thereby mediates the development of adaptive fast life-history traits that allow for earlier reproduction^19,21^. Our results partially support a hyporesponsive profile in high ELA participants, indicated by blunted HPA and PNS (re)activity but no differences in SNS regulation, at least when assessed using sAA. Thus, these systems may appear particularly sensitive when exposed to social threats early in life^19^. Moreover, ELA affects cerebral core regions of stress system regulation, i.e., the hippocampus, the amygdala and prefrontal cortex^39,51^. Exposure to ELA has been related to structural, functional and epigenetic changes in these regions^38^, which are in turn involved in ANS and HPA axis regulation^52^. Thus, ELA might affect both HPA axis and ANS stress responses via similar cerebral mechanisms.

Overall, we would like to emphasize that the effects of ELA reported here cannot be interpreted definitively as causal. Causal conclusions could only be drawn from longitudinal or experimental studies. There is evidence for such causal effects of ELA on stress regulation (e.g., HPA axis regulation) from experimental animal research and longitudinal prevention research with children^53–55^. With our cross-sectional design and the quasi-experimental categorisation of ELA groups (based on retrospective self-report), we must consider the observed changes in ELA rather as correlative than causal.

### Effects of early-life adversity and MS on stress reactivity

Our findings do not confirm an interaction between MS and ELA with regard to HPA axis reactivity, which led us to reject the second hypothesis and contrasts with the only prior study in this field^36^. Since this earlier study was conducted in males, differences between sexes might contribute to the diverging findings. In line with this explanation, sex has been suggested to moderate the effects of MS on behavioral and HPA axis responses^26^, with MS affecting cortisol stress responses only in males^34,35^. In contrast, we found an interaction between MS and MC in terms of PNS and subjective stress activation. Concerning the PNS, ELA individuals showed reduced HRV recovery from stress following contemplation on sleep (CS) compared to death (MS). Interestingly, parallel results occurred for subjective stress levels. High ELA individuals reported overall higher subjective stress when they had contemplated sleep (CS) compared to death (MS).

Bridging these results, an interplay between ELA and MS appears to be present in multiple stress systems. From a theoretical perspective, these findings might be interpreted as a successful psychophysiological adaptation to ELA^19^. Following the ACM, stress systems develop in a way to mediate adaptive responses under given environmental conditions. During sudden environmental changes, stress responses become temporarily maladaptive due to a mismatch with the new environmental conditions^19^. Thus, high ELA individuals might show adaptive responses under adverse conditions of acute mortality salience, indicated by fast PNS recovery and less subjective stress in the current study, while responses are less adaptive under deviating control conditions. Though speculative, the discussed cerebral alterations resulting from ELA provide a potential mechanism. Parts of these regions become activated also during MS^28,30^. Thus, acute threats such as MS might result in diverging activation patterns and subsequent outflow of stress systems, particularly in high ELA individuals. In sum, our findings suggest that death contemplation as a potentially soft form of acute threat, triggers highly sensitive and fast PNS and subjective responses, but does not require successive SNS and HPA axis responses in women.

### Limitations and future directions

A first limitation of this study refers to the generalizability of results, restricted to healthy, young women. With a female sample we intended to extent prior work, mainly conducted in males^36,40^. However, for a better understanding of the results at hand, a systematic examination of sex effects should be pursued in future studies. This was not feasible in the current study due to time and financial constraints. With the young sample (aged 18 to 30 years) we aimed to eliminate well-known age effects on HPA axis regulation, although we are aware that this also comes at the cost of limited generalization. Results might well differ in other age groups. Literature on the effect of both MS and ELA on psychobiological (re-)activity shows heterogeneous results with respect to age as potential moderating variable. A meta-analysis found that the effect size indicating blunted cortisol stress responses following early-life adversity was larger in adults than in children or adolescents^14^. Other meta-analyses did not reveal age a significant moderator of MS as well as ELA effects on (psycho-)biological responses^15,26^. Second, age and self-esteem were unequally distributed between the four groups and thus might have confounded our results. However, since we applied a narrow age range, and literature suggests that it is the early-life social environment that guides the development of self-esteem^56^, we do not expect our results to be causally driven by these variables. Further variables that differed between the four groups (e.g., depressiveness) did not change the significance of the results, when they were controlled for. However, we cannot exclude that additional variables that we did not assess might impact our current results. Third, data collection was conducted in two waves due to the Covid-19 pandemic. The pandemic constitutes a natural reminder of mortality^57^, with various negative health consequences^58^. Although we included cohort (prior, post onset of the pandemic) as a potential covariate in our statistical models, we cannot exclude that it has biased our results. Fourth, statistical power to detect a significant interaction effect was reduced due to unequal group sizes with a minimum of *n* = 14. An a posteriori power analysis indicated a power of 30.9% to detect a small interaction effect. Thus, replication in a balanced sample is needed. Finally, the methodological procedure was designed to test HPA axis responsivity as the primary outcome measure. To secondary explore SNS and PNS (re)activation, we applied common guidelines whenever possible. However, for specific ANS measurement, these results warrant further replication in future studies, especially when assessing the sympathetic branch of the ANS, where sAA might be a suboptimal marker^59,60^.

## Conclusions

The current findings highlight the multisystemic impact of low early-life MC on stress system regulation in young women, evident in reduced PNS and HPA axis (re)activity to acute stress. Dysregulations of stress systems are considered one possible biological pathway linking ELA with an increased risk for adverse health outcomes^61^. Identifying factors of intra- or interindividual variation in stress system (re)activity, such as MS, is thus of high scientific and therapeutic value. Besides methodological implications suggesting to account for the effect of MS in future studies, the current work provides insight into potential mechanisms that underlie the development of stress system dysregulation in adverse early-life environments.

## Methods

### Participants

Participants were recruited at the University and city of Konstanz via online and analogue advertisements. They first completed an online prescreening to determine (1) the extent of early-life MC using the Parental Bonding Instrument (PBI^42^; see section on psychological measures), and (2) study eligibility according to common exclusion criteria^62,63^. Exclusion criteria were an age < 18 or > 30 years, current pregnancy, symptoms of severe depression (BDI II sum score > 28;^64,65^), under- or overweight (body mass index (BMI) < 17.5 or > 30), smoking > 5 cigarettes per day, working night-shifts, current drug or medication intake affecting the cardiovascular or neuroendocrine system, self-reported current mental or physical (cardiovascular, metabolic, endocrine) disease, an increased risk for a Covid-19 infection (due to the university’s restrictions during the pandemic), and included only female sex. Eligible participants were invited to the laboratory and advised to attend sober (no food intake except water for 2 h, no alcohol intake for 18 h), and to refrain from smoking for 2 h and intense exercise the day before the session^62,63^.

The study was approved by the Ethics Committee of the University of Konstanz and follows the ethical principles of the Declaration of Helsinki (IRB statement 12/2017). Participants provided written informed consent and were compensated monetarily or by research credit hours for study participation of around 130 min.

### Procedure

Laboratory sessions were conducted in the afternoon at 0315 or 0500pm to maximize stress responsivity and control for circadian variations in baseline cortisol^66^. Due to the Covid-19 pandemic, sessions were conducted in two waves (Nov 2019-Feb 2020, Dec 2020-July 2021). Sessions were held in groups of two to four participants.

**Fig. 3:**
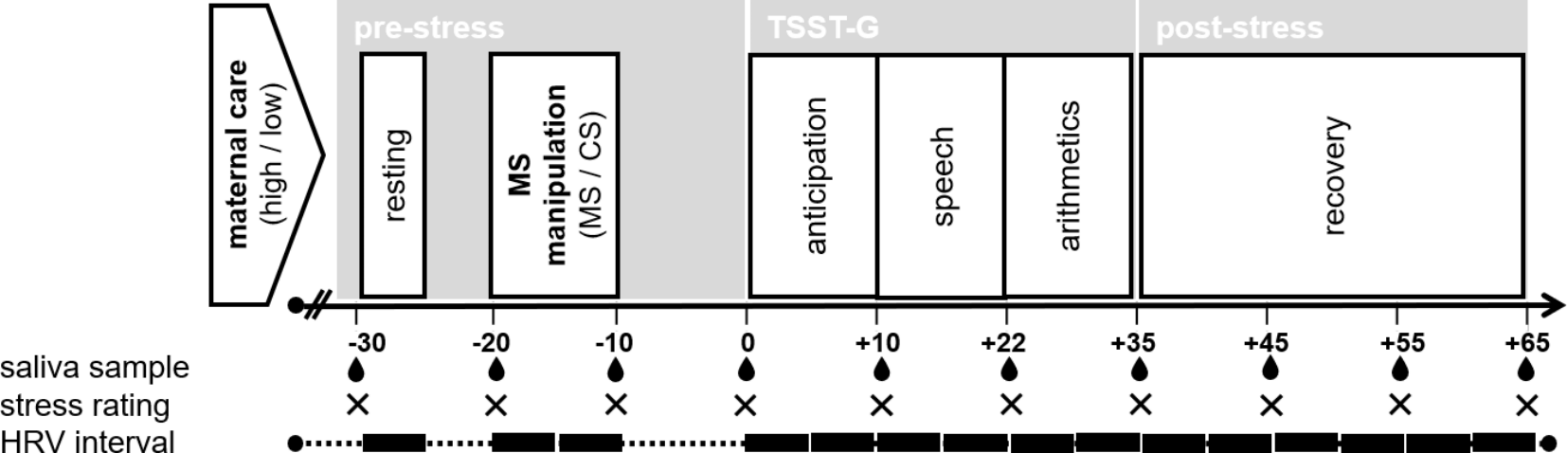
Study procedure. The experimental manipulation consisted of writing an essay regarding one’s own death (MS condition) or sleep (CS condition). Black boxes represent 5-min intervals for HRV calculation (I_1_ to I_15_). MS = mortality salience, CS = control stimulus, TSST-G = Trier Social Stress Test for groups, HRV = heart rate variability.

Each session consisted of three parts, including an initial resting and manipulation phase, an acute stress and final recovery phase^63^, see Fig. 3). After arrival, participants provided informed consent and were equipped with physiological measurement devices (see section on biological measures). For physiological baseline measurements, a first saliva sample was collected (-30 min), followed by a resting period of five minutes which participants spent sitting in an upright, motionless position. Next, for the MS manipulation participants reflected either on their death (MS condition), or on sleep (CS condition) for ten minutes (see subsequent section). After a short delay, they completed a manipulation check task and rested for a total of ten minutes. During the subsequent stress part participants underwent a modified version of the TSST-G. In the final recovery phase participants completed further questionnaires (see section on control variables). Sessions closed with a final saliva sample (+ 65 min), debriefing and compensation.

#### Experimental manipulation

The Mortality Attitudes Personality Survey (MAPS;^67^) was used as the most common MS manipulation to induce conscious thoughts of death^26^. It consists of two open-ended questions that ask for one’s emotions and thoughts when contemplating (1) the own death (MS condition), or (2) the neutral topic of sleep (CS condition). Participants answered the questions in the form of a short essay within 10 min. For the exact questions and German translations see supplemental material, S7.

##### Manipulation check

 Participants next answered a Word fragment Completion Task, a frequently applied method to check whether the MS manipulation increased death thought accessibility (e.g.,^68^). The WCT consisted of twenty word fragments each missing two letters to represent a meaningful word. Six were death-related fragments (e.g., STE _ _ EN) that could be completed to a death-related word (STERBEN (*die*)) or a neutral word (STELLEN (*put*)). Fourteen were death-unrelated filler fragments that could only be completed to neutral word(s). A WCT sum score was calculated as the number of death-related fragments completed in relation to death (range: 0–6). Higher WCT scores indicate more successful induction of death thoughts. Details on the German WCT and evaluation procedure are provided in the supplemental material, Table S8.

#### Stress induction

We used the TSST-G as a standardized laboratory paradigm to induce psychosocial stress in a group setting^43^, which was slightly adapted in terms of group sizes including up to four participants. It comprised an anticipation period (10 min), a stressful speech and mental arithmetics task (12 min each). Both stress tasks were performed in front of a white-clothed confederate panel (one woman, one man), who followed a neutral behavioral protocol. Participants were separated by mobile wall partitions and were called in random order to present. Details on the modified procedure are provided in the supplemental material, S9.

### Measures

#### Biological measures

##### Salivary cortisol and alpha amylase

Saliva samples for cortisol and alpha amylase analyses were collected using Salivettes (Sarstedt, Nümbrecht, Germany) at ten timepoints (-30, -20, -10, 0, + 12, +25, + 35, +45, + 55, +65 min in relation to stress onset; see Fig. 3). Samples were stored at -20 °C before analyses were conducted at the Biochemical laboratory of the neuropsychology group at of the University of Konstanz. For cortisol (nmol/l), a commercially available competitive enzyme immunosorbent assay was used (Cortisol Saliva ELISA, RE-52611, IBL International GmbH, Hamburg, Germany) with inter- and intra-assay coefficients of variance below 7.9% according to the manufacturer. For alpha amylase (U/ml), a commercially available enzymatic assay (alpha-Amylase Saliva assay, RE80111, IBL International GmbH, Hamburg, Germany) was applied. Intra- and inter-assay coefficients of variance were below 6.9% according to the manufacturer. sAA constitutes a reliable marker of SNS activity^59^.

##### Heart rate variability

Inter-beat-intervals (IBIs) were measured continuously with the mobile heart rate monitor device Polar H10 (Polar Electro Oy, Kempele, Finnland; sampling rate: 1000 Hz), and the application Heart Rate Variability Logger for iOS^69^, running on iPads, which were connected to the Polar sensors via Bluetooth. As part of the preprocessing procedure (see supplemental material, S10), HRV was calculated within fifteen time intervals of five minutes each (I_1_ to I_15_, see Fig. 3). As HRV parameter, we used the logarithmised high-frequency power band referred to as RSA as an index of PNS activity (i.e., specifically cardiac vagal tone)^63^.

#### Psychological measures

##### Maternal care

 The mother care subscale of the PBI (^42^; German version:^70^) was used as a retrospective self-report measure to assess perceived maternal care during the first 16 years of life. The twelve items of the subscale are answered on a 4-point Likert scale ranging from 0 (*very unlike*) to 3 (*very likely*). Higher sum scores (range: 0–36, cut-off: 27) indicate higher perceived early-life maternal care^42^. The mother care subscale was used during the prescreening to assign participants to either a high MC (score > 27) or low MC group (score < = 27).

##### Subjective stress

The Affect Grid (AG^71^), is a one-item measure to assess current affect. It consists of a 9 × 9 grid with the dimensions pleasure (horizontal) and arousal (vertical). To be answered, one point of the grid is selected. Besides singular scores for pleasure and arousal, a composite stress score can be derived^72^. Higher scores (range: 1–81) indicate higher levels of stress (i.e., lower levels of pleasure and higher levels of arousal)^73^. The AG was applied ten times concurrently to saliva sampling.

##### Control variables

 The following additional variables that impact HPA axis or ANS regulation^62,63^ were measured: self-reported age, BMI, last night sleep duration [min], mean daily exercising [min], religious identification, self-esteem using the Rosenberg Self-Esteem Scale (^74^; German version:^75^), symptoms of depression using the BDI-II (^64^; German version:^76^) and hormonal status (oral contraceptive use, follicular phase, luteal phase, unclear phase), which was determined based on self-reported duration and onset of last menstrual cycle^62^. Finally, we measured cohort (prior vs. post onset of the pandemic) as possible covariate. The effect of each variable was tested as part of the statistical analyses (see section below). For a full list of assessed psychological measures see https://osf.io/u2rq8/.

### Design and sample size

We implemented a between-subjects 2 *MC* (high, low) x 2 *mortality* condition (MS, CS) study design, resulting in four experimental groups. An a priori power analysis based on the second hypothesis was conducted using G*Power^77^. Results indicated a required sample size of *N* = 72 (*n* = 18 per group) to detect a group (between-subjects factor) by time (within-subjects factor) interaction effect of small to moderate size (*f* = 0.175) with a power of 95% (α = 0.05, *r* = .5 between repeated measures). The final sample comprised *N* = 74 participants; however, group sizes were unequal due to pandemic-related recruitment obstacles (see Table 1). Before statistical analyses, additional participants had to be excluded retrospectively due to (1) violation of inclusion criteria (*n* = 1), (2) technical problems with HRV recording (*n* = 3), (3) insufficient amount of saliva for biochemical sAA analyses (*n* = 5). Thus, analyses were in general based on *N* = 73 (for RSA: *N* = 70, for sAA: *N* = 68).

### Statistical analyses

Statistical analyses were conducted using R statistical software (version 4.0.4; R Core Team, 2021) and RStudio (version 2022.2.3.492; Rstudio Team, 2022), the packages ggplot2^78^, nlme^79^ and performance^80^. Prior to the analyses, data preprocessing included the transformation of cortisol (natural logarithm) and sAA levels (root transformation); for details see supplemental material, S10.

First, we performed multiple one-way Analyses of Variance (ANOVAs), and Fisher’s exact tests to test whether the four groups differed in possibly confounding variables (see section on control variables).

Second, we checked the success of the experimental MS and stress induction manipulations. To test whether the MS stimulus successfully increased death thought accessibility a non-parametric Kruskal-Wallis test with *mortality* as independent and the WCT score as dependent variable was applied. The success of the TSST-G to increase cortisol levels was tested as part of the subsequent growth curve model (GCM) approach.

Next, the hypothesized effects of *MC* and *mortality* on cortisol trajectories were tested using GCMs within the multilevel framework. Models to evaluate the effect of the TSST-G (unconditional model), *MC* (H1 model) and *mortality* (H2 model) were built hierarchically based on best model fit^81^: first, random intercepts (across individuals), fixed time trends (linear, quadratic, cubic), random slopes (across individuals), and a first-order autoregressive covariance structure (CAR) were incorporated (unconditional model); next, MC and MC by time interaction (H1 model) and mortality, mortality by time, MC by mortality and time by MC by mortality interaction (H2 model) were added as fixed effects. Time was entered as numeric predictor with the first included timepoint set to 0; since we were particularly interested in responses to stress, we only included the seven timepoints starting from stressor onset. MC (levels: 0 = low MC, 1 = high MC) and mortality (level: 0 = CS, 1 = MS) were entered as binary factors. We also re-run data analyses with MC as continuous predictor, with the results being provided in the supplemental material, S3. Nested models were compared pairwise with changes in model fit being assessed using the log-likelihood ratio and ANOVAs. For the final models with best model fit we report omnibus *F*-statistics; details on model comparison and final models’ beta coefficients are provided in the supplemental material. Finally, we also tested the impact of potential covariates (listed in the section on psychological measures). For this, each covariate and covariate by time interactions were added separately to the unconditional model as fixed effects. If a covariate led to a significant increase in model fit, we evaluated the hypothesized models based on this covariate model. In the results we then first report the final models without covariates, but (if present) also report and contrast models including significant covariates.

Finally, we explored the effects of MC and mortality on subjective stress, sAA and RSA trajectories using comparable GCMs. For RSA, we additionally calculated two change scores, to capture (1) RSA decrease under stress (RSA reactivity) and (2) RSA increase following stress (RSA recovery). RSA reactivity was computed as RSA during stress (mean of I_6_ to I_9_) minus RSA during baseline (I_1_); RSA recovery was computed as RSA following stress (I_10_) minus RSA during stress. Negative RSA reactivity scores indicate PNS withdrawal, positive RSA recovery scores indicate PNS re-dominance. Two 2 (MC) x 2 (mortality) ANOVAs with RSA reactivity respectively RSA recovery as dependent variables were used to separately test the effect of MC and mortality on these two components of PNS regulation.

Across analyses model assumptions were examined visually and statistically, using the performance package for GCMs^80^. Significant effects were followed up by Bonferroni-corrected post-hoc t-tests. Effect sizes are reported using eta squared and partial Cohen’s f. The level of significance was set to α = 0.05.

## Additional information

Supplementary information is provided online at https://osf.io/jfqey/ (Open Science Framework, Project DOI: https://doi.org/10.17605/OSF.IO/JFQEY). 

A preprint of this work has been published at https://osf.io/preprints/psyarxiv/merfa (PsyArXiv, Preprint DOI: 10.31234/osf.io/merfa). It has also been published as part of the first author´s dissertation at https://kops.uni-konstanz.de/entities/publication/70572d1f-50ec-480d-ab73-3fe390b220f1 (KOPS, the Institutional Repository of the University of Constance).

## Electronic supplementary material

Below is the link to the electronic supplementary material.

Supplementary Material 1

## Data Availability

The code for statistical analyses is available at https://osf.io/jfqey/ (Open Science Framework, Project DOI: https://doi.org/10.17605/OSF.IO/JFQEY). The data are available from the corresponding author upon request.

## References

[1] Ulrich-Lai, Y. M. & Herman, J. P. Neural regulation of endocrine and autonomic stress responses. *Nat. Rev. Neurosci.* **10**, 397–409 (2009). 19469025 10.1038/nrn2647 PMC4240627

[2] Joëls, M. & Baram, T. Z. The neuro-symphony of stress. *Nat. Rev. Neurosci.* **10**, 459–466 (2009). 19339973 10.1038/nrn2632 PMC2844123

[3] Leistner, C. & Menke, A. Hypothalamic-pituitary-adrenal axis and stress. *Handb. Clin. Neurol.* **175**, 55–64 (2020). 33008543 10.1016/B978-0-444-64123-6.00004-7

[4] Phillips, A. C., Ginty, A. T. & Hughes, B. M. The other side of the coin: Blunted cardiovascular and cortisol reactivity are associated with negative health outcomes. *Int. J. Psychophysiol.* **90**, 1–7 (2013). 23454029 10.1016/j.ijpsycho.2013.02.002

[5] Zorn, J. V. et al. Cortisol stress reactivity across psychiatric disorders: A systematic review and meta-analysis. *Psychoneuroendocrinology* **77**, 25–36 (2017). 28012291 10.1016/j.psyneuen.2016.11.036

[6] Turner, A. I. et al. Psychological stress reactivity and future health and disease outcomes: A systematic review of prospective evidence. *Psychoneuroendocrinology* **114**, 104599 (2020). 32045797 10.1016/j.psyneuen.2020.104599

[7] Agorastos, A., Pervanidou, P., Chrousos, G. P. & Baker, D. G. Developmental trajectories of early life stress and trauma: A narrative review on neurobiological aspects beyond stress system dysregulation. *Front. Psychiatry*. **10**, 118 (2019). 30914979 10.3389/fpsyt.2019.00118 PMC6421311

[8] Kalmakis, K. A. & Chandler, G. E. Adverse childhood experiences: Towards a clear conceptual meaning. *J. Adv. Nurs.* **70**, 1489–1501 (2014). 24329930 10.1111/jan.12329

[9] Berman, I. S. et al. Measuring early life adversity: A dimensional approach. *Dev. Psychopathol.* **34**, 499–511 (2022). 35314009 10.1017/S0954579421001826 PMC7613038

[10] Felitti, V. J. et al. Relationship of childhood abuse and household dysfunction to many of the leading causes of death in adults: The adverse childhood experiences (ACE) study. *Am. J. Prev. Med.* **14**, 245–258 (1998). 9635069 10.1016/s0749-3797(98)00017-8

[11] Smith, K. E. & Pollak, S. D. Rethinking concepts and categories for understanding the neurodevelopmental effects of childhood adversity. *Perspect. Psychol. Sci.* **16**, 67–93 (2021). 32668190 10.1177/1745691620920725 PMC7809338

[12] Petrullo, L. A., Mandalaywala, T. M., Parker, K. J., Maestripieri, D. & Higham, J. P. Effects of early life adversity on cortisol/salivary alpha-amylase symmetry in free-ranging juvenile rhesus macaques. *Horm. Behav.* **86**, 78–84 (2016). 27170429 10.1016/j.yhbeh.2016.05.004 PMC6719785

[13] Hosseini-Kamkar, N., Lowe, C. & Morton, J. B. The differential calibration of the HPA axis as a function of trauma versus adversity: A systematic review and p-curve meta-analyses. *Neurosci. Biobehav Rev.* **127**, 54–135 (2021). 33857580 10.1016/j.neubiorev.2021.04.006

[14] Bunea, I. M., Szentágotai-Tătar, A. & Miu, A. C. Early-life adversity and cortisol response to social stress: A meta-analysis. *Transl Psychiatry*. **7**, 1274 (2017). 29225338 10.1038/s41398-017-0032-3 PMC5802499

[15] Schaer, S., Muerner-Lavanchy, I., Schmidt, S. J., Koenig, J. & Kaess, M. Child maltreatment and hypothalamic-pituitary-adrenal axis functioning: A systematic review and meta-analysis. *Front. Neuroendocrinol.* **66**, 100987 (2022). 35202606 10.1016/j.yfrne.2022.100987

[16] Kirschbaum, C., Pirke, K. M. & Hellhammer, D. H. The ‘Trier Social stress test’ - a tool for investigating psychobiological stress responses in a laboratory setting. *Neuropsychobiology* **28**, 76–81 (1993). 8255414 10.1159/000119004

[17] Engert, V. et al. Perceived early-life maternal care and the cortisol response to repeated psychosocial stress. *J. Psychiatry Neurosci.* **35**, 370–377 (2010). 20964960 10.1503/jpn.100022 PMC2964367

[18] Pruessner, J. C., Champagne, F., Meaney, M. J. & Dagher, A. Dopamine release in response to a psychological stress in humans and its relationship to early life maternal care: A positron emission tomography study using [11 C]Raclopride. *J. Neurosci.* **24**, 2825–2831 (2004). 15028776 10.1523/JNEUROSCI.3422-03.2004 PMC6729514

[19] Del Giudice, M., Ellis, B. J. & Shirtcliff, E. A. The adaptive calibration model of stress responsivity. *Neurosci. Biobehav Rev.* **35**, 1562–1592 (2011). 21145350 10.1016/j.neubiorev.2010.11.007 PMC3068241

[20] Boyce, W. T. & Ellis, B. J. Biological sensitivity to context: I. An evolutionary-developmental theory of the origins and functions of stress reactivity. *Dev. Psychopathol.* **17**, 271–301 (2005). 16761546 10.1017/s0954579405050145

[21] Ellis, B. J., Figueredo, A. J., Brumbach, B. H. & Schlomer, G. L. Fundamental dimensions of environmental risk: The impact of harsh versus unpredictable environments on the evolution and development of life history strategies. *Hum. Nat.* **20**, 204–268 (2009). 25526958 10.1007/s12110-009-9063-7

[22] Lovallo, W. R. Early life adversity reduces stress reactivity and enhances impulsive behavior: Implications for health behaviors. *Int. J. Psychophysiol.* **90**, 8–16 (2013). 23085387 10.1016/j.ijpsycho.2012.10.006 PMC3587283

[23] McEwen, B. S. Stress, adaptation, and disease: Allostasis and allostatic load. *Ann. N Y Acad. Sci.* **840**, 33–44 (1998). 9629234 10.1111/j.1749-6632.1998.tb09546.x

[24] McEwen, B. S. & Akil, H. Revisiting the stress concept: Implications for affective disorders. *J. Neurosci.* **40**, 12–21 (2020). 31896560 10.1523/JNEUROSCI.0733-19.2019 PMC6939488

[25] McEwen, B. S. What is the confusion with cortisol? *Chronic Stress*. **3**, 1–3 (2019). 10.1177/2470547019833647 PMC6788742 31608312

[26] Burke, B. L., Martens, A. & Faucher, E. H. Two decades of terror management theory: A meta-analysis of mortality salience research. *Pers. Soc. Psychol. Rev.* **14**, 155–195 (2010). 20097885 10.1177/1088868309352321

[27] Greenberg, J., Pyszczynski, T., Solomon, S., Simon, L. & Breus, M. Role of consciousness and accessibility of death-related thoughts in mortality salience effects. *J. Pers. Soc. Psychol.* **67**, 627–637 (1994). 7965609 10.1037//0022-3514.67.4.627

[28] Klackl, J., Jonas, E. & Fritsche, I. Neural evidence that the behavioral inhibition system is involved in existential threat processing. *Soc. Neurosci.* **13**, 355–371 (2018). 28394199 10.1080/17470919.2017.1308880

[29] Poppelaars, E. S., Klackl, J., Scheepers, D. T., Mühlberger, C. & Jonas, E. Reflecting on existential threats elicits self-reported negative affect but no physiological arousal. *Front. Psychol.* **11**, 962 (2020). 32547446 10.3389/fpsyg.2020.00962 PMC7273972

[30] Jonas, E. et al. in Advances in experimental social psychology pp. 219–286. (2014).

[31] Griskevicius, V., Delton, A. W., Robertson, T. E. & Tybur, J. M. Environmental contingency in life history strategies: The influence of mortality and socioeconomic status on reproductive timing. *J. Pers. Soc. Psychol.* **100**, 241–254 (2011). 20873933 10.1037/a0021082 PMC3556268

[32] Tritt, S. M., Inzlicht, M. & Harmon-Jones, E. Toward a biological understanding of mortality salience (and other threat compensation processes). *Soc. Cogn.* **30**, 715–733 (2012).

[33] Buttlar, B., Walther, E., Pohl, C. & Gierens, A. Mind the gap between feeling bad and feeling dead: Stress but not death reminders elicit endocrine responses. *Death Stud.* **46**, 442–449 (2020). 32180538 10.1080/07481187.2020.1740829

[34] Byrd-Craven, J., Calvi, J. L. & Kennison, S. M. Rapid cortisol and testosterone responses to sex-linked stressors: Implications for the tend-and-befriend hypothesis. *Evol. Psychol. Sci.* **2**, 199–206 (2016).

[35] Byrd-Craven, J., Auer, B. J. & Kennison, S. M. Sex differences in salivary cortisol responses to sex-linked stressors: A test of the tend-and-befriend model. *Adapt. Hum. Behav. Physiol.* **1**, 408–420 (2015).

[36] Zakreski, E., Juster, R. P., Feneberg, A. C., Cooperman, C. & Pruessner, J. C. Inducing death thoughts reduces the Cortisol response to psychosocial stress similar to the effects of early-life adversity: A life-history perspective. *Adapt. Hum. Behav. Physiol.* **10**, 182–210 (2024).

[37] Sapolsky, R. M., Romero, L. M. & Munck, A. U. How do glucocorticoids influence stress responses? Integrating permissive, suppressive, stimulatory, and preparative actions. *Endocr. Rev.* **21**, 55–89 (2000). 10696570 10.1210/edrv.21.1.0389

[38] Heim, C., Entringer, S. & Buss, C. Translating basic research knowledge on the biological embedding of early-life stress into novel approaches for the developmental programming of lifelong health. *Psychoneuroendocrinology* **105**, 123–137 (2019). 30578047 10.1016/j.psyneuen.2018.12.011 PMC6561839

[39] Dedovic, K., Duchesne, A., Andrews, J., Engert, V. & Pruessner, J. C. The brain and the stress axis: the neural correlates of cortisol regulation in response to stress. *NeuroImage* **47**, 864–871 (2009). 19500680 10.1016/j.neuroimage.2009.05.074

[40] Bath, K. G. Synthesizing views to understand sex differences in response to early life adversity. *Trends Neurosci.* **43**, 300–310 (2020). 32353334 10.1016/j.tins.2020.02.004 PMC7195459

[41] Andrews, J., Ali, N. & Pruessner, J. C. Reflections on the interaction of psychogenic stress systems in humans: The stress coherence/compensation model. *Psychoneuroendocrinology* **38**, 947–961 (2013). 23522990 10.1016/j.psyneuen.2013.02.010

[42] Parker, G., Tupling, H. & Brown, L. B. A parental bonding instrument. *Br. J. Med. Psychol.* **52**, 1–10 (1979).

[43] von Dawans, B., Kirschbaum, C. & Heinrichs, M. The Trier Social stress test for groups (TSST-G): A new research tool for controlled simultaneous social stress exposure in a group format. *Psychoneuroendocrinology* **36**, 514–522 (2011). 20843608 10.1016/j.psyneuen.2010.08.004

[44] Sigrist, C. et al. Early life maltreatment and resting-state heart rate variability: A systematic review and meta-analysis. *Neurosci. Biobehav Rev.* **120**, 307–334 (2021). 33171141 10.1016/j.neubiorev.2020.10.026

[45] Lai, C. L. J., Lee, D. Y. H. & Leung, M. O. Y. Childhood adversities and salivary cortisol responses to the Trier Social stress test: A systematic review of studies using the children Trauma Questionnaire (CTQ). *Int. J. Environ. Res. Public. Health*. **18**, 29 (2020). 33374531 10.3390/ijerph18010029 PMC7793098

[46] McLaughlin, K. A., Sheridan, M. A., Humphreys, K. L., Belsky, J. & Ellis, B. J. The value of dimensional models of early experience: Thinking clearly about concepts and categories. *Perspect. Psychol. Sci.* **16**, 1463–1472 (2021). 34491864 10.1177/1745691621992346 PMC8563369

[47] Pollak, S. D. & Smith, K. E. Thinking clearly about biology and childhood adversity: Next steps for continued progress. *Perspect. Psychol. Sci.* **16**, 1473–1477 (2021). 34491865 10.1177/17456916211031539 PMC8564234

[48] Danese, A. & Widom, C. S. Objective and subjective experiences of child maltreatment and their relationships with psychopathology. *Nat. Hum. Behav.* **4**, 811–818 (2020). 32424258 10.1038/s41562-020-0880-3

[49] Thayer, J. F., Ahs, F., Fredrikson, M., Sollers, J. J. & Wager, T. D. A meta-analysis of heart rate variability and neuroimaging studies: Implications for heart rate variability as a marker of stress and health. *Neurosci. Biobehav Rev.* **36**, 747–756 (2012). 22178086 10.1016/j.neubiorev.2011.11.009

[50] Beauchaine, T. P. & Thayer, J. F. Heart rate variability as a transdiagnostic biomarker of psychopathology. *Int. J. Psychophysiol.* **98**, 338–350 (2015). 26272488 10.1016/j.ijpsycho.2015.08.004

[51] Lupien, S. J., McEwen, B. S., Gunnar, M. R. & Heim, C. Effects of stress throughout the lifespan on the brain, behaviour and cognition. *Nat. Rev.* **10**, 434–445 (2009). 10.1038/nrn2639 19401723

[52] Mueller, B., Figueroa, A. & Robinson-Papp, J. Structural and functional connections between the autonomic nervous system, hypothalamic-pituitary-adrenal axis, and the immune system: A context and time dependent stress response network. *Neurol. Sci.* **43**, 951–960 (2022). 35034231 10.1007/s10072-021-05810-1

[53] Cicchetti, D., Rogosch, F. A., Toth, S. L. & Sturge-Apple, M. L. Normalizing the development of cortisol regulation in maltreated infants through preventive interventions. *Dev. Psychopathol.* **23**, 789–800 (2011). 21756432 10.1017/S0954579411000307 PMC4191893

[54] Liu, D. et al. Maternal care, hippocampal glucocorticoid receptors, and hypothalamic-pituitary-adrenal responses to stress. *Science* **277**, 1659–1662 (1997). 9287218 10.1126/science.277.5332.1659

[55] Slopen, N., McLaughlin, K. A. & Shonkoff, J. P. Interventions to improve cortisol regulation in children: A systematic review. *Pediatrics* **133**, 312–326 (2014). 24420810 10.1542/peds.2013-1632 PMC3904273

[56] Orth, U., Robins, R. W. & Roberts, B. W. Low self-esteem prospectively predicts depression in adolescence and young adulthood. *J. Pers. Soc. Psychol.* **95**, 695–708 (2008). 18729703 10.1037/0022-3514.95.3.695

[57] Jutzi, C. A., Willardt, R., Schmid, P. C. & Jonas, E. Between conspiracy beliefs, ingroup bias, and system justification: How people use defense strategies to cope with the threat of COVID-19. *Front. Psychol.* **11**, 578586 (2020). 33101147 10.3389/fpsyg.2020.578586 PMC7555435

[58] Figueiredo, C. S. et al. COVID-19 pandemic impact on children and adolescents’ mental health: Biological, environmental, and social factors. *Prog Neuropsychopharmacol. Biol. Psychiatry*. **106**, 110171 (2021). 33186638 10.1016/j.pnpbp.2020.110171 PMC7657035

[59] Ali, N. & Nater, U. M. Salivary alpha-amylase as a biomarker of stress in behavioral medicine. *Int. J. Behav. Med.* **27**, 337–342 (2020). 31900867 10.1007/s12529-019-09843-x PMC7250801

[60] Bosch, J. A., Veerman, E. C. I., de Geus, E. J. & Proctor, G. B. Alpha-amylase as a reliable and convenient measure of sympathetic activity: Don’t start salivating just yet! *Psychoneuroendocrinology* **36**, 449–453 (2011). 21295411 10.1016/j.psyneuen.2010.12.019

[61] Sowder, K. L., Knight, L. A. & Fishalow, J. Trauma exposure and health: A review of outcomes and pathways. *J. Aggress. Maltreat Trauma.* **27**, 1041–1059 (2018).

[62] Strahler, J., Skoluda, N., Kappert, M. B. & Nater, U. M. Simultaneous measurement of salivary cortisol and alpha-amylase: Application and recommendations. *Neurosci. Biobehav Rev.* **83**, 657–677 (2017). 28864234 10.1016/j.neubiorev.2017.08.015

[63] Laborde, S., Mosley, E. & Thayer, J. F. Heart rate variability and cardiac vagal tone in psychophysiological research - recommendations for experiment planning, data analysis, and data reporting. *Front. Psychol.* **8**, 213 (2017). 28265249 10.3389/fpsyg.2017.00213 PMC5316555

[64] Beck, A. T., Steer, R. A. & Brown, G. K. *Beck Depression Inventory-II (BDI-II)* (The Psychological Corporation, 1996).

[65] Kühner, C., Bürger, C., Keller, F. & Hautzinger, M. Reliabilität Und Validität Des Revidierten Beck-Depressionsinventars (BDI-II). Befunde Aus Deutschsprachigen Stichproben. *Der Nervenarzt*. **78**, 651–656 (2007). 16832698 10.1007/s00115-006-2098-7

[66] Miller, R. et al. The CIRCORT database: Reference ranges and seasonal changes in diurnal salivary cortisol derived from a meta-dataset comprised of 15 field studies. *Psychoneuroendocrinology* **73**, 16–23 (2016). 27448524 10.1016/j.psyneuen.2016.07.201 PMC5108362

[67] Rosenblatt, A., Greenberg, J., Solomon, S., Pyszczynski, T. & Lyon, D. Evidence for Terror Management Theory: I. The effects of mortality salience on reactions to those who violate or uphold cultural values. *J. Pers. Soc. Psychol.* **57**, 681–690 (1989). 2795438 10.1037//0022-3514.57.4.681

[68] Chittaro, L., Sioni, R., Crescentini, C. & Fabbro, F. Mortality salience in virtual reality experiences and its effects on users’ attitudes towards risk. *Int. J. Hum. Comput. Stud.* **101**, 10–22 (2017).

[69] Altini, M. Heart rate variability logger (version 4.6.2) [IOS Mobile Application Software] (2013).

[70] Benz, A. B. E. et al. Psychometrische Kennwerte Einer Deutschen Übersetzung Des parental bonding instrument. *Psychother. Psychosom. Med. Psychol.* **72**, 34–44 (2022). 34255328 10.1055/a-1503-5328

[71] Russell, J. A., Weiss, A. & Mendelsohn, G. A. Affect Grid: A single-item scale of pleasure and arousal. *J. Pers. Soc. Psychol.* **57**, 493–502 (1989).

[72] Liapis, A., Katsanos, C., Sotiropoulos, D. G., Karousos, N. & Xenos, M. Stress in interactive applications: Analysis of the valence-arousal space based on physiological signals and self-reported data. *Multimed Tools Appl.* **76**, 5051–5071 (2017).

[73] Meier, M. et al. Effects of psychological, sensory, and metabolic energy prime manipulation on the acute endocrine stress response in fasted women. *Psychoneuroendocrinology* **134**, 105452 (2021). 34715529 10.1016/j.psyneuen.2021.105452

[74] Rosenberg, M. *Society and the Adolescent self-image* (Princeton University Press, 1965).

[75] von Collani, G. & Herzberg, P. Y. Zur Internen Struktur Des Globalen Selbstwertgefühls Nach Rosenberg. *Z. für Differentielle Und Diagnostische Psychologie*. **24**, 9–22 (2003).

[76] Hautzinger, M., Keller, F. & Kühner, C. *Beck Depressions-Inventar (BDI-II). Revision.* Harcourt Test Services., (2006).

[77] Faul, F., Erdfelder, E., Buchner, A. & Lang, A. G. Statistical power analyses using G*Power 3.1: Tests for correlation and regression analyses. *Behav. Res. Methods*. **41**, 1149–1160 (2009). 19897823 10.3758/BRM.41.4.1149

[78] Wickham, H. *ggplot2: Elegant Graphics for Data Analysis* (Springer-, 2016).

[79] Pinheiro, J., Bates, D., DebRoy, S. & Sarkar D. nlme: Linear and Nonlinear Mixed Effects Models (2021).

[80] Lüdecke, D., Ben-Shachar, M., Patil, I., Waggoner, P. & Makowski, D. Performance: An R package for assessment, comparison and testing of statistical models. *J. Open. Source Softw.* **6**, 3139 (2021).

[81] Bliese, P. D. & Ployhart, R. E. Growth modeling using random coefficient models: Model building, testing, and illustrations. *Organ. Res. Methods*. **5**, 362–387 (2002).

