# Supplementary material for: The effect of mortality salience and early-life maternal care on neuroendocrine, autonomic, and psychological stress responses
Source: Sci Rep. 2025 Jan 8;15:1349. doi: 10.1038/s41598-025-85380-w (PMC11711282; doi:10.1038/s41598-025-85380-w)
Supplement: Supplementary file 1 — Supplementary Material 1 [file 41598_2025_85380_MOESM1_ESM.docx]

Supplemental Material

The effect of mortality salience and early-life maternal care on neuroendocrine, autonomic and psychological stress responses

Ulrike U. Bentele*^1^, Paula Strobel^1^, Maria Meier^1,3^, Annika B. E. Benz^1^, Raphaela J. Gaertner^1^, Elea S. C. Klink^1^, Bernadette F. Denk^1,2^, Stephanie J. Dimitroff^4^, Eva Unternaehrer^1,3^, & Jens C. Pruessner^1,2^

^1^Department of Psychology, Division of Neuropsychology, University of Constance, Constance, Germany

^2^Centre for the Advanced Study of Collective Behaviour, University of Constance, Constance, Germany

^3^Child- and Adolescent Research Department, University Psychiatric Clinics Basel (UPK), University of Basel, Switzerland

^4^Department of Psychology, University of Montana, Montana 59812, United States

*Corresponding author:
Ulrike U. Bentele ([ulrike.bentele@uni-konstanz.de](mailto:ulrike.bentele@uni-konstanz.de))

ORCIDs:
Ulrike U. Bentele <https://orcid.org/0000-0002-3121-1957>
Maria Meier <https://orcid.org/0000-0002-1655-5479>
Jens C. Pruessner <https://orcid.org/0000-0002-8582-2980>

Eva Unternaehrer <https://orcid.org/0000-0002-3507-1883>

Table S1. Comparison of growth curve models for cortisol levels following step-wise incorporation of predictors (MC, mortality) using Analyses of Variance.

| Number | Model | Model specification |  | *df* | *logLikelihood* | Test | *df* | *Chi*^2^ | *p* |
| --- | --- | --- | --- | --- | --- | --- | --- | --- | --- |
|  |  | simpler model | added effect |  |  |  |  |  |  |
| 1 | FI |  |  | 2 | -591.58 |  |  |  |  |
| 2 | **RI** | FI | RI | 3 | -489.23 | 1 vs 2 | 1 | 204.69 | **<.001** |
| 3 | **Fixed.time** | RI | Time | 4 | -418.39 | 2 vs 3 | 1 | 141.68 | **<.001** |
| 4 | **Fixed.time^2^** | RI | Time^2^ | 5 | -398.68 | 3 vs 4 | 1 | 39.43 | **<.001** |
| 5 | **Fixed.time^3^** | RI | Time^3^ | 6 | -383.97 | 4 vs 5 | 1 | 29.41 | **<.001** |
| 6 | **RS** | Fixed.time^3^ | random Time | 8 | -271.55 | 5 vs 6 | 2 | 224.84 | **<.001** |
| 7 | **CAR** | RS | Covariance stucture | 9 | -256.64 | 6 vs 7 | 1 | 29.82 | **<.001** |
| 8 | MC | CAR | MC | 10 | -256.21 | 7 vs 8 | 1 | 0.86 | .354 |
| 9 | **MC x time^3^** | MC | Time^3^ x MC | 13 | -252.22 | 8 vs 9 | 3 | 7.98 | **.046** |
| 10 | mortality | MC x time^3^ | mortality | 14 | -251.22 | 9 vs 10 | 1 | 2.01 | .156 |
| 11 | mortality x time3 | mortality | Time^3^ x mortality | 17 | -249.91 | 10 vs 11 | 3 | 2.62 | .454 |
| 12 | mortality x MC | mortality x time^3^ | mortality x MC | 18 | -249.91 | 11 vs 12 | 1 | 0.00 | .946 |
| 13 | mortality x MC x time^3^ | mortality x MC | Time^3^ x mortality x MC | 21 | -247.62 | 12 vs 13 | 3 | 4.57 | .207 |

*Note.* Final models to evaluate the success of the stress induction (manipulation check), the effect of MC (hypothesis 1) and the interaction of MC and mortality (hypothesis 2). Cortisol levels were transformed using the natural logarithm. Significant improvement in model fit when models of increasing complexity were compared pairwise is printed in bold. *Time* represents the linear, *Time^2^* the quadratic, and *Time^3^* the cubic effect of time. Interactions are indicated by ‘x’. FI = Fixed intercept, RI = random intercept, RS = random slope, CAR = covariance structure, MC = maternal care. *N*_subjects_ = 73, *N*_observations_ = 511.

|  | Unconditional model | |  | H1 model |  |  | H2 model |  |  |
| --- | --- | --- | --- | --- | --- | --- | --- | --- | --- |
| *Fixed effects* | Estimate (*SE*) | *t* | *p* | Estimate (*SE*) | *t* | *p* | Estimate (*SE*) | *t* | *p* |
| Baseline | 1.33 (0.07) | 19.64 | **<.001** | 1.21(0.10) | 11.52 | **<.001** | 1.19 (0.15) | 7.83 | **<.001** |
| Time | 6.08 (0.78) | 7.77 | **<.001** | 4.42(1.21) | 3.66 | **<.001** | 6.47 (1.75) | 3.70 | **<.001** |
| Time^2^ | -2.60 (0.36) | -7.19 | **<.001** | -2.20(0.56) | -3.93 | **<.001** | -1.42 (0.81) | -1.76 | **<.079** |
| Time^3^ | -2.29 (0.31) | -7.47 | **<.001** | -1.49(0.47) | -3.15 | **.002** | -1.91 (0.69) | -2.76 | **.006** |
| MC |  |  |  | 0.22(0.14) | 1.60 | .115 | 0.09 (0.20) | 0.48 | .632 |
| Time x MC |  |  |  | 2.81(1.57) | 1.78 | .075 | -0.39 (2.25) | -0.17 | .863 |
| Time^2^ x MC |  |  |  | -0.69(0.73) | -0.94 | .347 | -1.03 (1.04) | -0.99 | .321 |
| Time^3^ x MC |  |  |  | -1.35(0.62) | -2.18 | .**030** | -0.75 (0.89) | -0.83 | .406 |
| mortality |  |  |  |  |  |  | 0.03 (0.21) | 0.14 | .893 |
| Time x mortality |  |  |  |  |  |  | -3.86 (2.39) | -1.61 | .107 |
| Time^2^ x mortality |  |  |  |  |  |  | -1.50 (1.10) | -1.36 | .174 |
| Time^3^ x mortality |  |  |  |  |  |  | 0.78 (0.95) | 0.82 | .412 |
| MC x mortality |  |  |  |  |  |  | 0.24 (0.27) | 0.89 | .375 |
| Time x MC x mortality |  |  |  |  |  |  | 6.09 (3.11) | 1.96 | .051 |
| Time^2^ x MC x mortality |  |  |  |  |  |  | 0.63 (1.44) | 0.44 | .660 |
| Time^3^ x MC x mortality |  |  |  |  |  |  | -1.13 (1.24) | -0.92 | .360 |
| *Random effects* | *SD* | correlation |  | *SD* | correlation |  | *SD* | correlation |  |
| Variance baseline | 0.42 | - |  | 0.43 | - |  | 0.43 | - |  |
| Variance slope | 0.01 | -0.07 |  | 0.01 | -0.10 |  | 0.01 | -0.18 |  |
| Residuals | 0.36 | - |  | 0.35 | - |  | 0.34 | - |  |

Table S2. Growth curve models predicting changes in cortisol levels by time (unconditional model), MC (H1 model) and condition (H2 model)

*Note. MC* (levels: 0 = low MC, 1 = high MC) and *mortality* condition (level: 0 = control stimulus, 1 = mortality salience) were entered as binary factor. *Time* represents the linear, *Time^2^* the quadratic, and *Time^3^* the cubic effect of time. Interactions are indicated by ‘x’. Cortisol levels were transformed using the natural logarithm. *N*_subjects_ = 73, *N*_observations_ = 511. MC = maternal care, *SE* = standard error, *SD* = standard deviation, H1 = Hypothesis 1, H2 = Hypothesis 2.

Table S3. Comparison of growth curve models for cortisol levels following step-wise incorporation of predictors (MC, mortality) using Analyses of Variance.

| Number | Model | Model specification |  | *df* | *logLikelihood* | Test | *df* | *Chi*^2^ | *p* |
| --- | --- | --- | --- | --- | --- | --- | --- | --- | --- |
|  |  | simpler model | added effect |  |  |  |  |  |  |
| 7 | **CAR** | RS | Covariance stucture | 9 | -256.64 | 6 vs 7 | 1 |  |  |
| 8 | MC | CAR | MC | 10 | -255.37 | 7 vs 8 | 1 | 2.55 | .110 |
| 9 | **MC x time^3^** | MC | Time^3^ x MC | 13 | -246.13 | 8 vs 9 | 3 | 18.47 | **<.001** |
| 10 | mortality | MC x time^3^ | mortality | 14 | -244.88 | 9 vs 10 | 1 | 2.51 | .113 |
| 11 | mortality x time3 | mortality | Time^3^ x mortality | 17 | -243.35 | 10 vs 11 | 3 | 3.06 | .382 |
| 12 | mortality x MC | mortality x time^3^ | mortality x MC | 18 | -243.34 | 11 vs 12 | 1 | 0.01 | .903 |
| 13 | mortality x MC x time^3^ | mortality x MC | Time^3^ x mortality x MC | 21 | -241.90 | 12 vs 13 | 3 | 2.88 | .410 |

*Note.* Stepwise modeling of cortisol trajectories starting from the unconditional model (CAR model) to evaluate the effect of *MC* (hypothesis 1) and the interaction of *MC* and *mortality* (hypothesis 2) on cortisol trajectories. Cortisol levels were transformed using the natural logarithm. Significant improvement in model fit when models of increasing complexity were compared pairwise is printed in bold. *Time^3^* represents the cubic effect of time. *MC* was added as continuous, *mortality* as binary predictor. Interactions are indicated by ‘x’. RS = random slope, CAR = covariance structure, MC = maternal care. *N*_subjects_ = 73, *N*_observations_ = 511.

Table S4. Comparison of growth curve models for stress levels following step-wise incorporation of predictors (MC, mortality) using Analyses of Variance.

| number | Model | Model specification |  | *df* | *logLikelihood* | Test | *df* | *Chi*^2^ | *p* |
| --- | --- | --- | --- | --- | --- | --- | --- | --- | --- |
|  |  | simpler model | added effect |  |  |  |  |  |  |
| 1 | FI |  |  | 2 | -2200.06 |  |  |  |  |
| 2 | **RI** | FI | RI | 3 | -2196.29 | 1 vs 2 | 1 | 7.53 | **.006** |
| 3 | **Fixed.time** | RI | Time | 4 | -2165.42 | 2 vs 3 | 1 | 61.75 | **<.001** |
| 4 | **Fixed.time^2^** | RI | Time^2^ | 5 | -2105.08 | 3 vs 4 | 1 | 120.68 | **<.001** |
| 5 | **Fixed.time^3^** | RI | Time^3^ | 6 | -2057.90 | 4 vs 5 | 1 | 94.36 | **<.001** |
| 6 | **RS** | Fixed.time^3^ | random Time | 8 | -2052.69 | 5 vs 6 | 2 | 10.42 | **.006** |
| 7 | **CAR** | RS | CAR | 9 | -2042.33 | 6 vs 7 | 1 | 20.73 | **<.001** |
| 8 | MC | CAR | MC | 10 | -2042.27 | 7 vs 8 | 1 | 0.12 | .729 |
| 9 | MC x time^3^ | MC | MC x Time^3^ | 13 | -2040.74 | 8 vs 9 | 3 | 3.05 | .384 |
| 10 | mortality | MC x time^3^ | mortality | 14 | -2040.53 | 9 vs 10 | 1 | 0.42 | .512 |
| 11 | mortality x time^3^ | mortality | Time^3^ x mortality | 17 | -2038.33 | 10 vs 11 | 3 | 4.41 | .221 |
| 12 | **mortality x MC** | mortality x time^3^ | mortality x MC | 18 | -2035.80 | 11 vs 12 | 1 | 5.07 | **.024** |
| 13 | mortality x MC x time^3^ | mortality x MC | Time^3^ x mortality x MC | 21 | -2035.51 | 12 vs 13 | 3 | 0.58 | .902 |

*Note.* Final models to evaluate the effect of MC and mortality. Significant improvement in model fit when models of increasing complexity were compared pairwise is printed in bold. *Time* represents the linear, *Time^2^* the quadratic and *Time^3^* the cubic effect of time. Interactions are indicated by ‘x’. FI = Fixed intercept, RI = random intercept, RS = random slope, CAR = covariance structure, MC = maternal care. *N*_subjects_ = 73, *N*_observations_ = 511.

Table S5. Comparison of growth curve models for sAA levels following step-wise incorporation of predictors (MC, mortality) using Analyses of Variance.

| number | Model | Model specification |  | *df* | *logLikelihood* | Test | *df* | *Chi*^2^ | *p* |
| --- | --- | --- | --- | --- | --- | --- | --- | --- | --- |
|  |  | simpler model | added effect |  |  |  |  |  |  |
| 1 | FI |  |  | 2 | -1401.39 |  |  |  |  |
| 2 | **RI** | FI | RI | 3 | -1269.37 | 1 vs 2 | 1 | 264.05 | **<.001** |
| 3 | **Fixed.time** | RI | Time | 4 | -1267.25 | 2 vs 3 | 1 | 4.22 | **.040** |
| 4 | **Fixed.time^2^** | RI | Time^2^ | 5 | -1200.02 | 3 vs 4 | 1 | 134.47 | **<.001** |
| 5 | **Fixed.time^3^** | RI | Time^3^ | 6 | -1173.31 | 4 vs 5 | 1 | 53.41 | **<.001** |
| 6 | RS | Fixed.time^3^ | random Time | 8 | -1172.44 | 5 vs 6 | 2 | 1.74 | .419 |
| 7 | **CAR** | Fixed.time^3^ | CAR | 7 | -1169.67 | 5 vs 7 | 1 | 7.30 | **.007** |
| 8 | MC | CAR | MC | 8 | -1169.66 | 7 vs 8 | 1 | 0.02 | .888 |
| 9 | MC x time^3^ | MC | MC x Time^3^ | 11 | -1168.71 | 8 vs 9 | 3 | 1.89 | .595 |
| 10 | mortality | MC x time^3^ | mortality | 12 | -1168.68 | 8 vs 10 | 1 | 0.07 | .798 |
| 11 | mortality x time^3^ | mortality | Time^3^ x mortality | 15 | -1167.28 | 10 vs 11 | 3 | 2.79 | .426 |
| 12 | mortality x MC | mortality x time^3^ | mortality x MC | 16 | -1166.80 | 11 vs 12 | 1 | 0.97 | .324 |
| 13 | mortality x MC x time^3^ | mortality x MC | Time^3^ x mortality x MC | 19 | -1165.95 | 12 vs 13 | 3 | 1.69 | .640 |

*Note.* Final models to evaluate the effect of MC and mortality. Significant improvement in model fit when models of increasing complexity were compared pairwise is printed in bold. *Time* represents the linear, *Time^2^* the quadratic and *Time^3^* the cubic effects of time. Interactions are indicated by ‘x’. FI = Fixed intercept, RI = random intercept, RS = random slope, CAR = covariance structure, MC = maternal care. *N*_subjects_ = 68, *N*_observations_ = 476.

Table S6. Comparison of growth curve models for RSA levels following step-wise incorporation of predictors (MC, mortality) using Analyses of Variance.

| number | Model | Model specification |  | *df* | *logLikelihood* | Test | *df* | *Chi*^2^ | *p* |
| --- | --- | --- | --- | --- | --- | --- | --- | --- | --- |
|  |  | simpler model | added effect |  |  |  |  |  |  |
| 1 | FI |  |  | 2 | -1400.41 |  |  |  |  |
| 2 | **RI** | FI | RI | 3 | -1115.79 | 1 vs 2 | 1 | 569.23 | **<.001** |
| 3 | **Fixed.time** | RI | Time | 4 | -1111.30 | 2 vs 3 | 1 | 8.99 | **.003** |
| 4 | **Fixed.time^2^** | RI | Time^2^ | 5 | -1051.70 | 3 vs 4 | 1 | 119.20 | **<.001** |
| 5 | **Fixed.time^3^** | RI | Time^3^ | 6 | -1033.54 | 4 vs 5 | 1 | 36.32 | **<.001** |
| 6 | RS | Fixed.time^3^ | random Time | 8 | -1031.96 | 5 vs 6 | 2 | 3.16 | .206 |
| 7 | **AR** | RS | AR | 7 | -958.22 | 5 vs 7 | 1 | 150.63 | **<.001** |
| 8 | **MC** | RS | MC | 8 | -955.23 | 7 vs 8 | 1 | 5.98 | **.015** |
| 9 | MC x time^3^ | MC | MC x Time^3^ | 11 | -954.19 | 8 vs 9 | 3 | 2.08 | .557 |
| 10 | mortality | MC x time^3^ | mortality | 12 | -954.19 | 9 vs 10 | 1 | 0.00 | .986 |
| 11 | mortality x time^3^ | mortality | Time^3^ x mortality | 15 | -953.98 | 10 vs 11 | 3 | 0.41 | .937 |
| 12 | mortality x MC | mortality x time^3^ | mortality x MC | 16 | -953.98 | 11 vs 12 | 1 | 0.00 | .967 |
| 13 | mortality x MC x time^3^ | mortality x MC | Time^3^ x mortality x MC | 19 | -952.59 | 12 vs 13 | 3 | 2.79 | .425 |

*Note.* Final models to evaluate the effect of MC and mortality. Significant improvement in model fit when models of increasing complexity were compared pairwise is printed in bold. *Time* represents the linear, *Time^2^* the quadratic and *Time^3^* the cubic effects of time. Interactions are indicated by ‘x’. FI = Fixed intercept, RI = random intercept, RS = random slope, AR = covariance structure, MC = maternal care. *N*_subjects_ = 70, *N*_observations_ = 1050.

S7 Experimental manipulation with German translation in italics

MS items (Mortality Attitudes Personality Survey (MAPS, 1))

Briefly describe the emotions that the thought of your own death arouses in you.

*Bitte beschreiben Sie kurz die Emotionen, welche Gedanken an den eigenen Tod bei Ihnen auslösen.*

Jot down, as specifically as you can, what you think will happen to you as you physically die and once you are physically dead

*Bitte notieren Sie stichwortartig und möglichst präzise Ihre Gedanken dazu, was mit Ihrem Körper geschehen wird, wenn Sie sterben und nachdem Sie gestorben sind.*

Control items

Briefly describe the emotions that the thought of sleeping induces in you.

*Bitte beschreiben Sie kurz die Emotionen, welche Gedanken an Schlaf bei Ihnen auslösen.*

Jot down, as specifically as you can, what you think happens to you as you fall asleep and once you are sleeping.

*Bitte notieren Sie stichwortartig und möglichst präzise Ihre Gedanken dazu, was mit Ihnen geschieht, wenn Sie einschlafen und nachdem Sie eingeschlafen sind.*

S8 Word fragment Completion Task (WCT)

A German version of a WCT was established following previously described procedures [1,2]: First, we created a list of 31 death-related words (e. g. Grab (*tomb*); [1,3]). Next, fragments of these words were tested within an online pilot study. Participants (*N*=18 (female=12, male=5, diverse=1), *M*_age_=27.44, *SD*_age_ = 10.72, range=22-58 years) were asked to complete each of the displayed word fragments to a meaningful German word. Word fragments were considered as suitable death-related words for the WCT if they were completed in death-related manner not more than 50% of time [2]. The final WCT comprised 20 word fragments including six death-related and fourteen neutral filler words.

German Word completion Task (WCT) including fragmented and full words

| Item number | Word fragment | Word category | Full word |
| --- | --- | --- | --- |
| 1 | BR _ _ | Filler | Brei (*mash*) |
| 2 | STE _ _ EN | Death-related | sterben (*die*),  stellen (*put*) |
| 3 | _ _ CKE | Filler | Jacke (*jacket*) |
| 4 | TA _ _ E | Filler | Tasse (*cup*) |
| 5 | FA_ _ EN | Filler | fallen (*fall*) |
| 6 | GR _ _ | Death-related | Grab (*tomb*),  Grat (*ridge*) |
| 7 | SCH _ _ LL | Filler | schnell (*fast*) |
| 8 | M _ _D | Death-related | Mord (*murder*),  Mund (*mouth*) |
| 9 | _ _ NNE | Filler | Sonne (*sun*) |
| 10 | WI _ _ E | Filler | Wiege (*cradle*) |
| 11 | _ _ NGER | Filler | Finger (*finger*) |
| 12 | SA _ _ | Death-related | Sarg (*coffin*),  Salz (*salt*) |
| 13 | SCHU _ _ | Filler | Schule (*school*) |
| 14 | _ _ CH | Filler | Buch (*book*) |
| 15 | EN _ _ | Death-related | Ende (*end*),  Ente (*duck*) |
| 16 | PA _ _EN | Filler | passen (*suit*) |
| 17 | ST _ _L | Filler | Stuhl (*chair*) |
| 18 | _ _ UFT | Death-related | Gruft (*crypt*),  Kluft (*gulf*) |
| 19 | AU _ _ | Filler | Auto (*car*) |
| 20 | L _ _ SE | Filler | leise (*quiet*) |

Note. For neutral filler words one out of multiple possible solution is presented. The English translation of the full words is shown in brackets.

S9 Psychosocial stress induction

We applied a modified version of the TSST-G to induce psychosocial stress in a group setting [4]. It slightly differed from the original protocol in terms of time duration and group sizes.

The modified version consisted of an anticipation period (10min), a stressful public speaking and mental arithmetics task (12min each). During anticipation participants received instructions and prepared for the speech task, before they were brought to a second room. Both subsequent tasks were guided and performed in front of a white-coated committee of confederates (one women, one man), who followed a neutral, standardized behavioral protocol. The speech task consisted of a fake job interview; the second unprepared task required participants to count back in steps of 17 from a given four-digit number. Participants were separated by mobile wall partitions and called in random order to present. Between both tasks, the experimenter entered the room for saliva sampling; after task cessation participants were guided back to the testing room. The TSST-G was conducted in groups of four participants (54.5% of session), with each participant working three minutes on each task. In case of no-shows groups of three participants (27.3% of sessions) with four minutes working time per participant and task, or two participants (18.2% of sessions) with five minutes per participant and task plus a 1-min break in between were tested [5,6].

S10 Data preprocessing

*Heart rate variability (HRV).* Preprocessing of recorded Inter-beat-intervals (IBIs) was done using R statistical software (see 2.5) and in house scripts for data cleaning and processing. First, artifacts in raw data were removed, if differences between consecutive IBIs exceeded a visually defined threshold (20-45%); missing IBIs were then imputed by the means of surrounding IBIs. Next, fifteen time intervals (I_1_ to I_15_) of five minutes each were defined [7]: baseline (5min), manipulation (2 x 5min), TSST anticipation (2 x 5min), TSST public speaking (2 x 5min), TSST mental arithmetics (2 x 5min) and recovery (6 x 5min) (see Fig. 1). For each interval, absolute power within the high-frequency band (0.15-0.4Hz; HF-HRV) was determined using the R package RHRV [8]. Thereby, high-frequency power was calculated as the mean of power values within five 1-min time intervals. Finally, RSA was determined as the natural logarithm of high-frequency power [9].

*Data preprocessing prior to statistical analyses.* Repeated measures of cortisol, sAA, RSA and subjective stress were preprocessed as follows. First, missing values were *replaced* to allow for subsequent statistical analyses (cortisol: 8 of 730 data points, sAA: 3 of 680 data points, RSA: 0 of 1050 data points, subjective stress: 0 of 730 data points). For cortisol and amylase, single missing values (a) at the first timepoint were replaced by the sample’s mean at that timepoint, (b) at the sample’s peak were replaced by the individual’s peak value, (c) at all other timepoints were interpolated linearly. Consecutively missing values at the first and second timepoint were replaced by the sample’s mean at the respective timepoints. For subjective stress levels and RSA no missing values occurred. Second, values that exceeded the sample’s mean ± 3 SD at a respective timepoint (limit value) were *winsorized* to this limit value to reduce the impact of statistical outliers (cortisol: 11 of 730 data points, sAA: 6 of 680 data points, RSA: 7 of 1050 data points, subjective stress: 6 of 730 data points). Finally, to use parametric tests skewed data were transformed. Cortisol levels were transformed using the natural logarithm; sAA levels were root transformed. For subjective stress levels no appropriate transformation was identified to improve skewedness, which is why statistical analyses were conducted with untransformed values.

References

1. Greenberg, J., Pyszczynski, T., Solomon, S., Simon, L. & Breus, M. Role of consciousness and accessibility of death-related thoughts in mortality salience effects. *J. Pers. Soc. Psychol.* **67,** 627–637 (1994).

2. Arndt, J., Greenberg, J. & Cook, A. Mortality salience and the spreading activation of worldview-relevant constructs: Exploring the cognitive architecture of terror management. *J. Exp. Psychol. Gen.* **131,** 307–324 (2002).

3. Mikulincer, M. & Florian, V. Exploring individual differences in reactions to mortality salience: Does attachment style regulate terror management mechanisms? *J. Pers. Soc. Psychol.* **79,** 260–273 (2000).

4. von Dawans, B., Kirschbaum, C. & Heinrichs, M. The Trier Social Stress Test for Groups (TSST-G): A new research tool for controlled simultaneous social stress exposure in a group format. *Psychoneuroendocrinology* **36,** 514–522 (2011).

5. Bentele, U. U. *et al.* The impact of maternal care and blood glucose availability on the cortisol stress response in fasted women. *Journal of neural transmission* **128,** 1287–1300 (2021).

6. Meier, M. *et al.* Effects of psychological, sensory, and metabolic energy prime manipulation on the acute endocrine stress response in fasted women. *Psychoneuroendocrinology* **134,** 105452 (2021).

7. Quintana, D. S., Alvares, G. A. & Heathers, J. A. J. Guidelines for Reporting Articles on Psychiatry and Heart rate variability (GRAPH): recommendations to advance research communication. *Transl. Psychiatry* **6,** e803 (2016).

8. Rodriguez-Linares, L. *et al.* RHRV: Heart Rate Variability Analysis of ECG Data. (2020).

9. Laborde, S., Mosley, E. & Thayer, J. F. Heart rate variability and cardiac vagal tone in psychophysiological research - Recommendations for experiment planning, data analysis, and data reporting. *Front. Psychol.* **8,** 213 (2017).
